# Bowel ultrasound measurements in healthy children — systematic review and meta-analysis

**DOI:** 10.1007/s00247-019-04567-2

**Published:** 2019-12-14

**Authors:** Elsa A. van Wassenaer, Floris A. E. de Voogd, Rick R. van Rijn, Johanna H. van der Lee, Merit M. Tabbers, Faridi S. van Etten-Jamaludin, Angelika Kindermann, Tim G. J. de Meij, K. B. Gecse, Geert R. D’Haens, Marc A. Benninga, Bart G. P. Koot

**Affiliations:** 1 Pediatric Gastroenterology, Emma Children’s Hospital, Amsterdam UMC, University of Amsterdam, Meibergdreef 9, 1105 AZ Amsterdam, The Netherlands; 2 Gastroenterology and Hepatology, Amsterdam UMC, University of Amsterdam, Amsterdam, The Netherlands; 3 Radiology, Amsterdam UMC, University of Amsterdam, Amsterdam, The Netherlands; 4 Pediatric Clinical Research Office, Emma Children’s Hospital, Amsterdam UMC, University of Amsterdam, Amsterdam, The Netherlands; 5 Knowledge Institute of the Dutch Association of Medical Specialists, Utrecht, the Netherlands; 6 Medical Library, Amsterdam UMC, University of Amsterdam, Amsterdam, The Netherlands; 7 Pediatric Gastroenterology Emma Children’s Hospital, Amsterdam UMC, Vrije Universiteit Amsterdam, Amsterdam, The Netherlands

**Keywords:** Bowel, Children, Meta-analysis, Normal subjects, Systematic review, Ultrasound

## Abstract

**Background:**

Ultrasound (US) is a noninvasive method of assessing the bowel that can be used to screen for bowel pathology, such as Inflammatory Bowel Disease, in children. Knowledge about US findings of the bowel in healthy children is important for interpreting US results in cases where disease is suspected.

**Objective:**

To assess the bowel wall thickness in different bowel segments in healthy children and to assess differences in bowel wall thickness among pediatric age categories.

**Materials and methods:**

We conducted a systematic search in the PubMed, Embase, Cochrane, and CINAHL databases for studies describing bowel wall thickness measured by transabdominal US in healthy children. We excluded studies using contrast agent. We calculated the pooled mean and standard deviation scores and assessed differences among age categories (0–4 years, 5–9 years, 10–14 years, 15–18 years), first with analysis of variance (ANOVA) and further with subsequent Student’s *t*-tests for independent samples, corrected for multiple testing.

**Results:**

We identified 191 studies and included 7 of these studies in the systematic review. Reported bowel wall thickness values ranged from 0.8 mm to 1.9 mm in the small bowel and from 1.0 mm to 1.9 mm in the colon. The mean colonic bowel wall thickness is larger in children ages 15–19 years compared to 0–4 years (range in difference: 0.3–0.5 mm [corrected *P*<0.02]).

**Conclusion:**

The reported upper limit of bowel wall thickness in healthy children is 1.9 mm in the small bowel and the colon, and mean thickness increases slightly with age in jejunum and colon. These values can be used as guidance when screening for bowel-related pathology in children.

**Electronic supplementary material:**

The online version of this article (10.1007/s00247-019-04567-2) contains supplementary material, which is available to authorized users.

## Introduction

Ultrasound (US) is a noninvasive and safe method of imaging the bowel, which makes it suitable for use in children. Bowel US can be used to screen for bowel-related pathology in children, mostly inflammatory bowel disease [1]. Features of inflammation — most important of which is increased bowel wall thickness but also increased vascularity and presence of enlarged lymph nodes — can be detected by US with high specificity [2]. However, to interpret US results, it is important to understand normal findings and age-related changes in healthy children. To gain more knowledge about the ultrasonographic appearance of the bowel in healthy children, we performed a systematic review of the literature describing US of the bowel in healthy children. The aim of this systematic review was to assess the mean and range of the bowel wall thickness in all different bowel segments in healthy children. The secondary objectives were to assess differences in bowel wall thickness among age categories and to describe other reported ultrasonographic findings in healthy children, such as presence of visible lymph nodes.

## Materials and methods

### Search strategy

We conducted a systematic search with help of a clinical librarian (F.S.E.-J.) in the PubMed, Embase (Ovid), Cochrane Library, and CINAHL (EBSCO) databases for studies describing bowel wall thickness measured by transabdominal US in healthy subjects aged 0–18 years. We excluded studies using contrast agent, studies only describing the appendix and studies whose full text was unavailable. Additionally we excluded articles not written in English, French, German, Spanish, Italian or Dutch. We did not restrict our search to a certain period of time.

The search terms are shown in the supplementary material. The titles and abstracts of the articles retrieved using the search strategy were screened independently by two reviewers (E.A.W., F.A.E.V., each with 4 years of experience in bowel ultrasound) to identify potentially eligible studies. The same reviewers then retrieved full texts of these potentially eligible studies and independently assessed them for eligibility. Any disagreements were resolved through discussion with a third reviewer (B.G.P.K. with 20 years of experience in pediatric gastroenterology).

### Data extraction

We used a standardized piloted form to extract data from the included studies and to assess methodological quality. Extracted information included number of patients, demographic details, study design, location of participant recruitment, definition of “healthy” as defined by authors, US technique (brand, probe, bowel preparation, method of bowel wall measurement) and bowel wall thickness per segment (jejunum; ileum; cecum; ascending, transverse and descending colon; rectum) per age category in millimeters (mm). Age was categorized as follows: 0–4 years, 5–9 years, 10–14 years and 15–18 years, based on an earlier study [3].

### Methodological quality

To assess methodological quality we used the Checklist for Cross-Sectional/Prevalence Studies from the Agency for Healthcare Research and Quality Methodology [4] and added three questions deemed relevant by the reviewers, based on recommendations from the *Cochrane Handbook for Systematic Reviews on Diagnostic Test Accuracy* [5]: “Did test operators have appropriate training?” and “Was ultrasound technique described properly?” and “Was definition of healthy clearly described?” Methodological quality was independently assessed by two reviewers (E.A.W. and F.A.E.V.).

### Data analysis

To calculate the mean bowel wall thickness per segment per age category over studies, we performed a meta-analysis. Studies that used the standard way of measuring bowel wall thickness (from the serosa/muscularis propria interface to the mucosa/lumen interface) were included in the meta-analysis. The sample-size weighted pooled mean and pooled standard deviation (SD) scores were calculated with Excel version 2016 (Microsoft, Redmond, WA). We first assessed the differences among the age categories with ANOVA. If a significant difference was found, we further investigated with subsequent Student’s *t*-tests for independent samples, corrected for multiple testing with the Bonferroni method using GraphPad Prism® version 7 (GraphPad, San Diego, CA). First, we investigated differences between consecutive age categories, and if no significant differences between consecutive age groups were found, differences between other age categories were analyzed. In the Results sections, only the corrected *P*-values are presented.

## Results

### Included studies

**Fig. 1:**
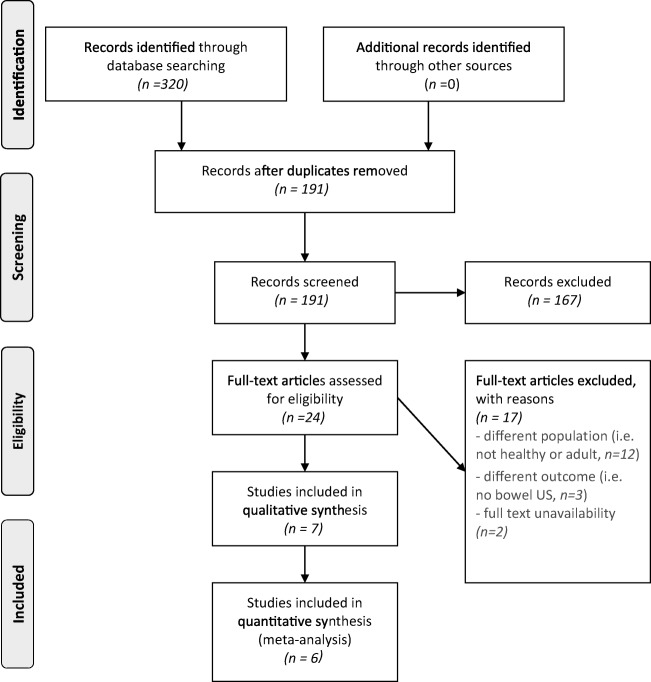
PRISMA (Preferred Reporting Items for Systematic Reviews and Meta-Analyses) flowchart. *US* ultrasound

After removing duplicates, we identified 191 records. After screening title/abstracts, we excluded 167 studies and checked 24 full-text articles (Fig. 1). Reasons for exclusion were inclusion of a different population (i.e. not healthy or adult, *n*=12); use of a different outcome (e.g., no bowel US, *n*=3); and full-text unavailability (*n*=2: one article was never published as a digital version, and the authors of the other article did not respond to our inquiry). Finally, we included seven studies in this systematic review. Study characteristics are depicted in Table 1.

**Table 1:** Characteristics of included studies

Author (country)	*n*	Age (years)	Brand US	Frequency + transducer	Method of measuring BWT	Bowel preparation	Segments evaluated
Chiorean et al. 2014 (UK) [1]	58	0–18	Siemens Elegra, Acuson Sequoia	5–15 MHz, curved-array and linear transducers	Measurement from just above first mucosal interface	6 h of fasting	Ileum, cecum, colon
Connett et al. 1999 (UK) [6]	9	5–13	Not reported	5–7.5 MHz, curved and linear transducers	Measurement of 3-layer structure	Not reported	Ascending, transverse, descending colon^a^
Epifanio et al. 2011 (Brazil) [7]	17	0–0.5	Philips	Linear transducer, frequency unknown	Measurement from mucosal layer till serosa	3 h of fasting	Jejunum, ileum, and ascending and descending colon
Haber and Stern 2000 (Germany) [3]	86	0–18	Acuson 128	7-MHz linear-array transducer	Measurement from hypoechoic mucosa and lamina propria to hypoechoic muscularis propria	None	Jejunum, ileum, colon
Pohl et al. 1997 (Germany) [8]	12	0–18	Acuson 128	5-MHz linear transducer	Not reported	None	Jejunum, ileum, and ascending and descending colon
Ramsden et al. 1998 (UK) [9]	12	3–12	Siemens Quantum	8-MHz curvilinear transducer	Measurement between the echogenic luminal contents and the outer border of the echo-poor muscle	None	Ascending, transverse, descending colon
Robinson et al. 2004 (USA) [10]	13	0–1	ATL 5000 (Bothell) or Sequoia (Acuson)	7.5–12 MHz, transducer unknown	Measurement from the outer wall to outer wall diameter of compressed loop divided by two	Not reported	Ileum

*BWT* bowel wall thickness, *h* hours

^a^Only mean colonic wall thickness is presented, not per segment

### Methodological quality

The methodological quality assessment is presented in Table 2. Six of the seven studies reported the technique used for the ultrasound and measurements. However, in most articles the methodological quality of several other important features of the assessment was unclear or low. Three studies defined their inclusion and exclusion criteria for the healthy control group and three reported a clear definition of “healthy” for participants. The definitions used for healthy were “asymptomatic” [7, 10], “not known to have any gastrointestinal disease” [9], “attending outpatient clinic with minor orthopedic problems” [6] or no definition [2, 8]. In one study a variety of diagnoses was included, such as psychogenic abdominal pain, familial growth retardation and previous urinary tract infection [3]. Most studies (*n*=4) did not report any measures for quality assurance, such as assessing intra- or interobserver agreement. In only one of the included studies a second ultrasound was performed on a subsequent day in a subset of children, and the researchers found no significant difference in measurements. Most studies (*n*=5) did not report whether the operator had appropriate training.

**Table 2:** Methodological quality assessment

Author, year	Did test operators have appropriate training?	Did authors list inclusion and exclusion criteria?	Did authors indicate time period used for identifying patients?	Did authors indicate whether or not subjects were consecutive?	Did authors state their definition of healthy?	Did authors describe any assessments undertaken for quality assurance purposes ?	Did authors explain any patient exclusions from analysis?	Did authors explain how missing data were handled in the analysis?	Was ultrasound technique described properly?
Chiorean et al. 2014 [1]	UNCLEAR	HIGH	LOW	HIGH	HIGH	HIGH	LOW	LOW	LOW
Connett et al. 1999 [9]	UNCLEAR	UNCLEAR	UNCLEAR	UNCLEAR	UNCLEAR	UNCLEAR	UNCLEAR	UNCLEAR	LOW
Epifanio et al. 2011 [7]	LOW	LOW	LOW	LOW	LOW	HIGH	LOW	LOW	LOW
Haber and Stern 2000 [3]	UNCLEAR	LOW	LOW	UNCLEAR	UNCLEAR	LOW	UNCLEAR	UNCLEAR	LOW
Pohl et al. 1997 [10]	UNCLEAR	HIGH	UNCLEAR	UNCLEAR	UNCLEAR	HIGH	UNCLEAR	UNCLEAR	UNCLEAR
Ramsden et al. 1998 [8]	UNCLEAR	UNCLEAR	UNCLEAR	UNCLEAR	LOW	LOW	UNCLEAR	UNCLEAR	LOW
Robinson et al. 2004 [6]	LOW	LOW	UNCLEAR	LOW	LOW	HIGH	LOW	UNCLEAR	LOW

*HIGH* high concerns about quality, *LOW* no concerns about quality, *UNCLEAR* quality unclear

### Ultrasound technique

Two of the included studies used a bowel preparation protocol: 3–6 h of fasting. Two studies did not report a specific bowel preparation protocol and three did not prepare the bowel before US examination. All of the included studies used linear probes (5–12 MHz) to measure bowel wall thickness. Most studies measured bowel wall thickness from the serosa/muscularis propria interface to the mucosa/lumen interface; however one study measured the complete diameter of a compressed bowel loop (i.e. two bowel walls combined) and divided this by two. This article was not incorporated in the meta-analysis [10].

### Bowel wall thickness

**Fig. 2:**
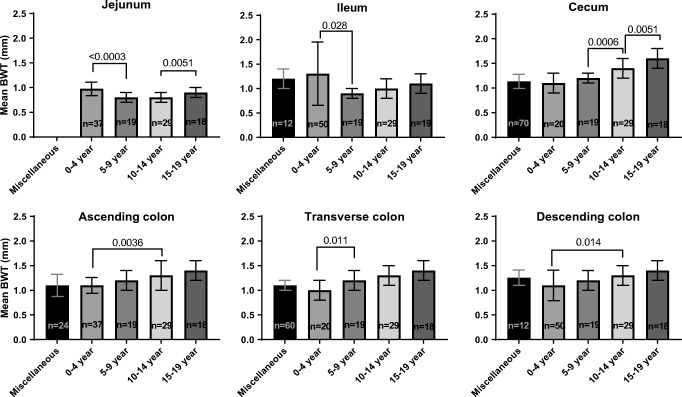
Mean bowel wall thickness (BWT) in millimeters (mm) per age category, displayed per segment. Differences were tested with analysis of variance and subsequently with Student’s *t*-tests for independent samples. *P*-values were corrected for multiple testing with the Bonferroni method. Miscellaneous: age range 3–18 years

The reported results of all included studies are presented in Table 3, and the pooled mean bowel wall thickness per age category and per segment is presented in Table 4 and Fig. 2. Bowel wall thickness was measured in the jejunum (*n*=3 studies, 115 participants), ileum (*n*=3 studies, 173 participants), cecum (*n*=4 studies, 156 participants), ascending colon (*n*=4 studies, 124 participants), transverse colon (*n*=3 studies, 156 participants), and descending colon (*n*=5 studies, 283 participants). One study described measurements in the colon without specifying the segment [6]. No study measured bowel wall thickness in the rectum. The bowel wall thickness in the jejunum, ileum, cecum and colon ranged from 0.5 mm to 1.1 mm, 0.6 mm to 1.9 mm, 0.7 mm to 1.9 mm, and 0.7 mm to 1.9 mm, respectively. In the study that used a different measurement method and included infants aged 0–13 months, the mean (standard deviation [SD]) ileal and terminal ileal bowel wall thicknesses were 2.0±1.0 mm and 2.8±0.8 mm, respectively [10].

**Table 3:** Reported bowel wall thickness per segment and age category in mm

Author	Age (years)	Jejunum (SD) [range]	Ileum (SD) [range]	Cecum (SD) [range]	Ascending colon (SD) [range]	Transverse colon (SD) [range]	Descending colon (SD) [range]
Chiorean et al. [1] (*n*=58)	11±4	–	1.0 (0.1) [0.9–1.30]	1.1 (0.1) [0.9–1.3]	–	1.1 (0.1) [0.9–1.3]	1.3 (0.1) [1.2–1.7]
Connett et al. [6] (*n*=9)	5–13	–	–	0.9 (0.1) [0.7–1.3]^a^			
Epifanio et al. [7] (*n*=17)	0–0.5	1.3 (0.3) [−]^b^	1.9 (0.5) [−]^b^	–	1.1 (0.1) [−]^b^	–	1.2 (0.4) [−]^b^
Haber and Stern [3] (*n*=20, *n*=19, *n*=29, *n*=18)	0–4	0.7 (0.1) [0.6–0.8]	0.8 (0.1) [0.6–1.0]	1.1 (0.2) [0.7–1.3]	1.1 (0.2) [0.7–1.3]	1.0 (0.2) [0.6–1.2]	1.1 (0.2) [0.8–1.4]
	5–9	0.8 (0.1) [0.5–1.0]	0.9 (0.1) [0.7–1.1]	1.2 (0.1) [1.0–1.5]	1.2 (0.2) [0.8–1.6]	1.2 (0.2) [0.8–1.5]	1.2 (0.2) [0.8–1.4]
	10–14	0.8 (0.1) [0.6–1.1]	1.0 (0.2) [0.7–1.5]	1.4 (0.2) [1.0–1.9]	1.3 (0.3) [0.6–1.8]	1.3 (0.2) [0.7–1.7]	1.3 (0.2) [0.8–1.9]
	15–19^c^	0.9 (0.1) [0.6–1.0]	1.1 (0.1) [1.0–1.4]	1.6 (0.2) [1.3–1.8]	1.4. (0.2) [1.1–1.9]	1.4 (0.2) [0.9–1.6]	1.4 (0.2) [1.1–1.7]
Pohl et al. [8] (*n*=12)	9 ± −	1.2 (0.2) [−]^b^	–	1.3 (0.2) [−]^b^	1.2 (0.2) [−]^b^	–	1.2 (0.2) [−]^b^
Ramsden et al. [9] (*n*=12)	3–12	–	–	–	1.0 (−)^b^ [0.6–1.2]	1.1 (−)^b^ [0.9–1.3]	1.1 (−)^b^ [0.8–1.4]
Robinson et al. [10] (*n*=13)	0–1	–	2.0 (1.1) [−]^b,d^	–	–	–	–

*SD* standard deviation

^a^Specific segment not reported

^b^– not reported

^c^Number of 19-year-old subjects unknown

^d^Used a different measurement method, see Table 1

**Table 4:** Pooled mean bowel wall thickness per segment and age category in mm

Age category (years)	Jejunum (SD) [*n*]	Ileum (SD) [*n*]	Cecum (SD) [*n*]	Ascending colon (SD) [*n*]	Transverse colon (SD) [*n*]	Descending colon (SD) [*n*]
0–4	1.0 (0.4) [37]	1.3 (0.6) [37]	1.1 (0.2) [20]	1.1 (0.2) [37]	1.0 (0.2) [20]	1.1 (0.2) [37]
5–9	0.8 (0.1) [19]	0.9 (0.1) [19]	1.1 (0.1) [19]	1.1 (0.2) [19]	1.2 (0.2) [19]	1.2 (0.2) [19]
10–14	0.8 (0.1) [29]	1.0 (0.2) [29]	1.4 (0.2) [29]	1.3 (0.3) [29]	1.3 (0.2) [29]	1.3 (0.2) [29]
15–19^a^	0.9 (0.1) [18]	1.1 (0.1) [18]	1.6 (0.2) [18]	1.4 (0.2) [18]	1.4 (0.2) [18]	1.4 (0.2) [18]
Miscellaneous^b^	–	1.2 (0.2) [11]	1.1 (0.2) [70]	1.1 (0.2) [24]	1.1 (0.1) [70]	1.3 (0.2) [82]

*SD* standard deviation

^a^Number of 19-year-old subjects unknown

^b^Age range: 3–18 years

### Influence of age

Four studies did not report bowel wall thickness measurements in specific age categories. The results of these studies are depicted in Table 4 and Fig. 2 as “miscellaneous.” Age in these studies ranged from 3 years to 18 years and bowel wall thickness ranged from 0.6 mm to 1.7 mm. Three studies reported bowel wall thickness measurements in children aged 0–4 years [3, 7, 10], and one study measured bowel wall thickness in children aged 5–9 years, 10–14 years and 15–19 years [3]; in both jejunum and ileum, the mean bowel wall thickness was higher in the youngest age group compared to the older age groups (0.18 mm difference in jejunum, *P*<0.0003; 0.40 mm difference in ileum, *P*=0.028). In the colon, the mean bowel wall thickness increased slightly with age in every segment. The differences in mean bowel wall thickness between the children aged 0–4 years and 15–19 years were 0.5 mm, 0.3 mm, 0.4 mm and 0.2 mm in the cecum and the ascending, transverse and descending colon, respectively (all *P*<0.01).

### Other sonographic findings

One study described the presence of mesenteric lymph nodes in the ileocecal region in 62–69% of healthy controls [10]. The subjects in this study were aged 0–13 months and the lymph nodes, measured at the longest axis, had a mean (SD) diameter of 8 (3.4) mm. The same study also described the presence of free intraperitoneal fluid in 3/13 of subjects (23%). Another study quantified the presence of mesenteric vessels in healthy children aged 0–6 months in ileal and jejunal regions [7] and found 4/17 (23.5%) to have increased vascularity, defined as >12% vessel density in a 4-cm^2^ area measured with color Doppler US.

## Discussion

In this systematic review we assessed the bowel wall thickness as measured with ultrasound in healthy children. Obtaining reference values from a healthy population is of great importance because the role of bowel US in children is rapidly increasing [12]. We found that the reported values of bowel wall thickness in healthy children range from 0.8 mm to 1.9 mm in small bowel and from 1.0 mm to 1.9 mm in the colon, when measuring from the serosa/muscularis propria interface to the mucosa/lumen interface. Although all included studies had some methodological flaws, these values can be used as guidance in clinical practice when screening children suspected of having bowel pathology, especially inflammatory bowel disease.

In this systematic review, we also found a difference in bowel wall thickness among pediatric age categories: colonic bowel wall thickness was larger in older compared to younger children. The differences between the youngest age groups (0–4 years) and the oldest age groups (15–19 years) ranged from 0.3 mm to 0.5 mm. This is in line with a study in 122 healthy adults aged 23–79 years [11] that also found a positive correlation between age and bowel wall thickness (r=0.069, *P*=0.003). This raises the question whether ultrasonographers should use different cut-off values for different age categories.

A study in children who were newly diagnosed with Crohn's disease (aged 9–18 years) reported an ileal bowel wall thickness of 5.6±1.8 mm [13]. In addition, a study in children aged 2–18 with active ulcerative colitis reported colonic bowel wall thickness values of >3 mm [14]. Hence, the small difference in bowel wall thickness between older and younger children is probably not clinically relevant in the diagnosis and follow-up of children with inflammatory bowel disease, also because children with inflammatory bowel disease are usually in their teens [15]. However, for children with early onset inflammatory bowel disease this needs to be confirmed because there are no data on US findings in this patient group.

The relevance of this age-related bowel wall thickness difference for the diagnostics in other causes of enterocolitis, like allergic or infectious causes, is unclear because there is a scarcity of data on US findings in these disorders. Interestingly, bowel wall thickness in the small bowel was larger in children aged 0–4 years compared to children aged 5–9 years, in both the jejunum and the ileum. Also, the weighted pooled SD in the ileum was quite high (0.6 mm) in the children aged 0–4 years. Two of the included studies also reported the presence of mesenteric lymph nodes, free fluid and increased mesenteric vascularity in the ileal and jejunal regions in children aged 0–1 years [7, 10]. This implies that among infants the small bowel wall is variable because of changes in lymphoid tissue in the Peyer patch associated with immunologic maturation and that reference values for small bowel wall thickness in this group of children have a wide range, possibly affecting the accuracy for individual patients. However, the small study populations of the included studies should be taken into account when interpreting these results.

The studies included in this systematic review used different approaches for bowel preparation. Some experts advise not to use any preparation, or merely to take in non-carbonated fluid 30 min before the US examination [16], while a recent consensus statement of the European Society of Paediatric Radiology (ESPR) and European Society of Gastrointestinal and Abdominal Radiology (ESGAR) states that children should not eat any solid food or drink carbonated fluid or milk for 2–6 h before bowel US exam, based on expert opinion [17]. Nylund et al. [11] compared bowel wall thickness measured after overnight fasting to bowel wall thickness measured 30 min after eating a 300 Kcal meal in 23 healthy adults and reported a small increase of bowel wall thickness in the terminal ileum (change from 1.1±0.2 mm to 1.2±0.2 mm, *P*<0.05) and sigmoid colon (change from 1.2±0.3 mm to 1.4±0.4 mm, *P*<0.05) [11]. Although the second measurement was not blinded and this is a small difference, it seems advisable to standardize bowel preparation protocols, especially in research settings. The same study compared bowel wall thickness measured with 8-MHz transducers to 12-MHz transducers using mixed linear model analysis and found a small influence of transducer type, with lower bowel wall thickness measurements when using the 12-MHz transducer (−0.05 mm, *P*<0.001). In this systematic review the included studies used different types of transducers, which is most likely explained by the year in which the studies were conducted; older studies used lower-frequency transducers. We do not think that the currently presented results are influenced by this small difference of 0.05 mm, but for future studies on bowel wall thickness, it would be advisable to uniformly use high-frequency transducers to minimize measurement variation.

This systematic review shows that all studies on bowel US in healthy children have some methodological flaws. First, the methodological quality of most included studies was unclear or low on important features of the methodological quality assessment. Examples of this are the unclear protocols for missing data and unclear or absent measures for quality assurance, such as intra-operator reliability analyses. Another limitation of the included studies is the small number of patients in the age categories 5–9 years, 10–14 years and 15–19 years. Only one of the included studies reported the bowel wall thickness in these age categories [3], and the others either included only infants [7, 10] or presented only the results for all participants together, regardless of their age [2, 6, 8, 9]. In addition, not all studies used a clear definition of “healthy children,” although most studies did report an absence of gastrointestinal symptoms.

To generate reference values, future studies should be strict on inclusion and exclusion criteria and use a clear definition of “healthy,” use protocolled bowel preparation and uniformly use high-frequency probes. Bowel wall thickness should be measured separately in each segment and in different age categories, whereby it would be worth a consideration splitting the youngest age groups, taking into account the results of the studies in infants presented in this systematic review. In addition, it would be of value to investigate the presence of other ultrasonographic markers of inflammation in healthy children, such as increased vascularity of the bowel wall, and presence of lymph nodes.

## Conclusion

We found that maximal reported bowel wall thickness in healthy children is 1.9 mm in small bowel and in colon. Furthermore we found that the range in ileal bowel wall thickness in healthy infants is larger than in older children and that in the colon the bowel wall thicknesses increase with age. This small age-dependent difference in colonic bowel wall thickness is not clinically relevant for assessing bowel disease in pediatric inflammatory bowel disease, and values for bowel wall thickness reported in this systematic review can be used as guidance when screening for bowel-related pathology. However, for the development of strict reference values of bowel wall thickness in healthy children, larger studies with strict methodology are needed.

## Electronic Supplementary Material

ESM 1 (DOCX 13 kb)
