# Supplementary material for: Bowel ultrasound measurements in healthy children — systematic review and meta-analysis
Source: Pediatr Radiol. 2019 Dec 14;50(4):501–8. doi: 10.1007/s00247-019-04567-2 (PMC7067709; doi:10.1007/s00247-019-04567-2)
Supplement: Supplementary file 1 — (DOCX 13 kb) [file 247_2019_4567_MOESM1_ESM.docx]

# Supplementary material

**Search strategy**

**PubMed**

147 hits:

(bowel wall[tiab] OR intestinal wall [tiab] OR colonic wall[tiab] OR ileal wall[tiab] OR ileum wall[tiab]) AND ("Ultrasonography"[Mesh] OR ultraso*[tiab] OR sono*[tiab] OR echo*[tiab]) AND ("Pediatrics"[Mesh] OR "Child"[Mesh] OR "Adolescent"[Mesh] OR child* OR adolescen* OR teen* OR youth[tiab] OR young[tiab] OR pediatric*[tiab] OR paediatric*[tiab]) AND ("Comparative Study" [Publication Type] OR compar*[tiab] OR healthy[tiab] OR normal[tiab] OR disease free[tiab] OR non-disease*[tiab] OR school child*[tiab] OR schoolchild*[tiab]) 

**Embase (Ovid)**:

123 hits

| **#** | **Searches** | **Results** |
| --- | --- | --- |
| 1 | (bowel wall or intestinal wall or colonic wall or ileal wall or ileum wall).ti,ab,kw. | 11254 |
| 2 | exp echography/ or (ultraso* or sono* or echo*).ti,ab,kw. | 1048789 |
| 3 | exp pediatrics/ or child/ or adolescent/ or (child* or adolescen* or teen* or youth or young or pediatric* or paediatric*).ti,ab,kw. | 3732338 |
| 4 | exp comparative study/ or (compar* or healthy or normal or disease free or non-disease* or school child* or schoolchild*).ti,ab,kw. | 9019943 |
| 5 | 1 and 2 and 3 and 4 | 210 |
| 6 | limit 5 to conference abstract status | 87 |
| 7 | 5 not 6 | 123 |

**Cochrane Library**

18 hits

ID Search Hits

#1 bowel wall or intestinal wall or colonic wall or ileal wall or ileum wall:ti,ab,kw (Word variations have been searched) 524

#2 ultraso* or sono* or echo*:ti,ab,kw (Word variations have been searched) 41469

#3 child* or adolescen* or teen* or youth or young or pediatric* or paediatric*:ti,ab,kw (Word variations have been searched) 261098

#4 #1 and #2 and #3 18

**CINAHL (Ebsco)**

32 hits

TI ( bowel wall or intestinal wall or colonic wall or ileal wall or ileum wall ) OR AB ( bowel wall or intestinal wall or colonic wall or ileal wall or ileum wall )

AND

(MH "Ultrasonography+") OR ( TI ( ultraso* or sono* or echo* ) OR AB ( ultraso* or sono* or echo* ) )

AND

(MH "Pediatrics+") OR (MH "Child+") OR (MH "Adolescence+") OR ( TI ( child* or adolescen* or teen* or youth or young or pediatric* or paediatric* ) OR AB ( child* or adolescen* or teen* or youth or young or pediatric* or paediatric* ) )

AND

(MH "Comparative Studies") OR TI ( compar* or healthy or normal or disease free or non-disease* or school child* or schoolchild* ) OR AB ( compar* or healthy or normal or disease free or non-disease* or school child* or schoolchild* )
